# Bronchus-associated lymphoid tissue in lung transplantation: a facilitator of rejection or regulator of tolerance?

**DOI:** 10.3389/fimmu.2025.1553533

**Published:** 2025-02-05

**Authors:** Alexander N. Wein, Charles R. Liu, Daniel Kreisel

**Affiliations:** ^1^ Department of Pathology and Immunology, Washington University School of Medicine, St. Louis, MO, United States; ^2^ Department of Surgery, Washington University School of Medicine, St. Louis, MO, United States

**Keywords:** bronchus-associated lymphoid tissue (BALT), lung transplantation, rejection, tolerance, FoxP3+ regulatory T cells

## Abstract

The role of bronchus-associated lymphoid tissue (BALT) in the regulation of immune responses to transplanted lungs remains an area of interest and controversy. Early studies in a rat pulmonary transplant model suggested BALT may accelerate rejection of grafts by inducing a local and systemic inflammatory response. Such observations were corroborated in intrapulmonary tracheal transplant models in the rat. While some human studies have described the presence of BALT in grafts that have been chronically rejected, others did not observe an association between induction of BALT and adverse outcomes. More recent investigations have found that BALT, enriched in immunoregulatory cell populations, is induced in tolerant mouse lung allografts, suggesting that such structures may be protective against rejection. Thus, the role of BALT in lung transplantation biology is complex. Insights gained from studies that focus on the role of BALT in lung transplantation may be harnessed to develop new therapies.

## Introduction

Lung transplantation is the definitive treatment for end-stage pulmonary disease. Unfortunately, lung transplantation has the worst survival out of all solid organ transplants with a median survival of approximately 6 years (1). Most lung grafts fail due to the development of chronic lung allograft dysfunction (CLAD), a pathogenic process characterized by the fibrotic remodeling of the airways, interstitium and pleura (2). CLAD manifests as either an obstructive phenotype known as bronchiolitis obliterans syndrome (BOS), with fibrosis or loss of small airways, or a restrictive phenotype known as restrictive allograft syndrome (RAS), which can be characterized by a spectrum of histological features including acute presentations of diffuse alveolar damage and intra-alveolar fibrinous exudates, which can progress to chronic end-stage fibrosis and pleuroparenchymal fibroelastosis (3–5). Mechanisms resulting in CLAD development are multifactorial, and factors that lead to BOS or RAS remain incompletely understood. Therefore, elucidating mechanisms that regulate pulmonary allograft rejection and tolerance is critical to developing therapies which prolong the survival of lung transplant recipients.

A key area of research in the study of lung transplant immunology is bronchus-associated lymphoid tissue (BALT), a pulmonary tertiary lymphoid organ (TLO) which was originally thought to contribute to rejection but has more recently been demonstrated to play a critical role in maintaining graft tolerance. BALT contributes to pulmonary immune defense by acquiring antigens from the airway, priming T and B cells, and maintaining immunologic memory (6). BALT is constitutive in some mammalian species (e.g., rabbits and rats) where it plays an essential role in pulmonary immune defense, while it is induced in other species (e.g., humans and mice) in response to inflammation or infection and may be referred to as inducible BALT (iBALT) (7). In humans, the development of iBALT, which can form in peribronchial or perivascular areas, can be associated with various inflammatory conditions, such as chronic obstructive pulmonary disease or rheumatoid arthritis (8–11). BALT has been classically described as having B cell follicles with follicular dendritic cells (FDCs), segregated B cell and T cell zones, and high endothelial venules (HEVs), which facilitate leukocyte trafficking to and from BALT (6). Importantly, HEVs in BALT express peripheral node addressin (PNAd), a feature unique to secondary and tertiary lymphoid organs (6). However, iBALT often lacks these organizational features, suggesting that the term BALT may be broadly applicable to pulmonary lymphoid aggregates with PNAd^+^ HEVs (6, 12).

**Figure 1:**
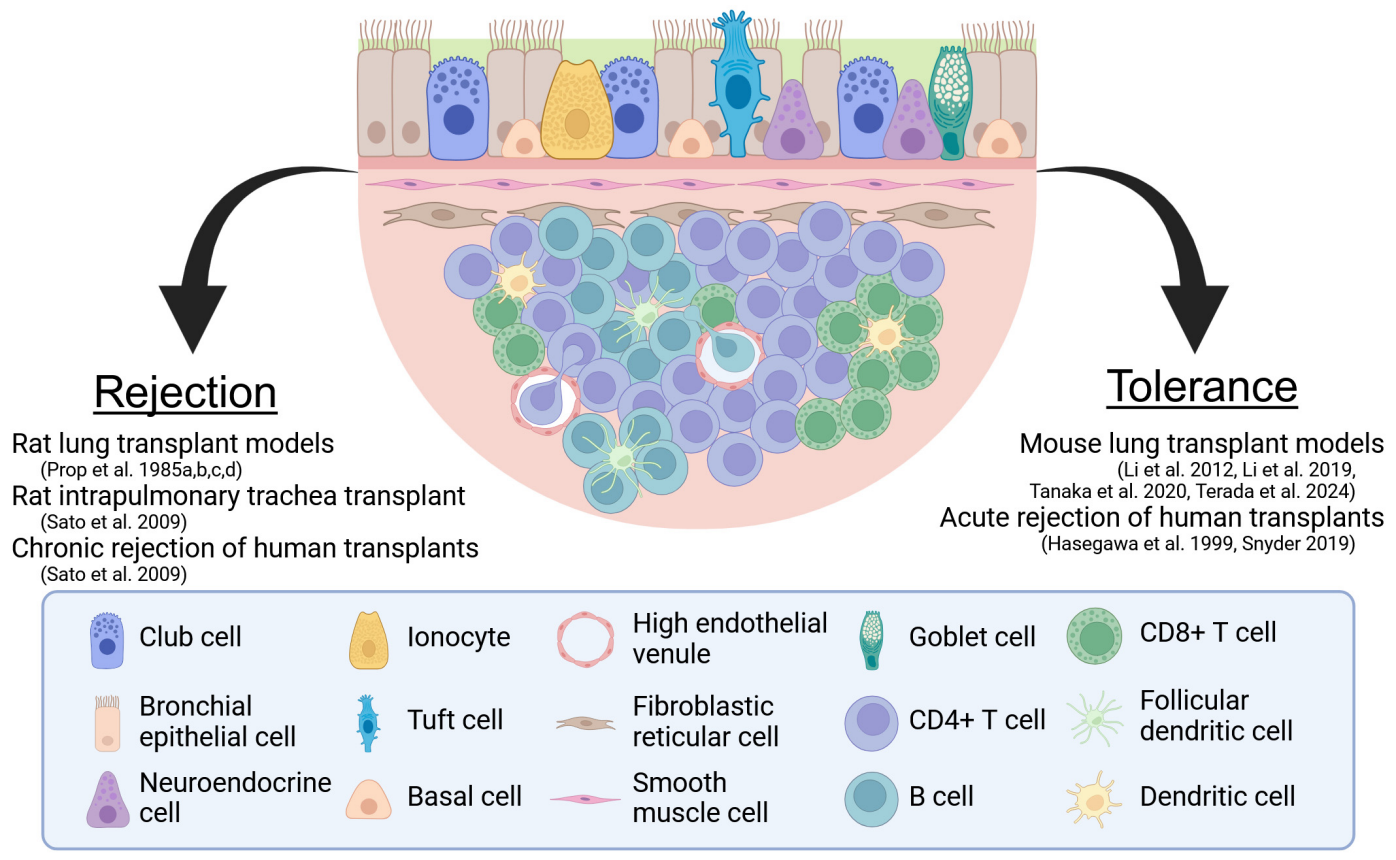
The two sides of BALT. A model BALT is shown with the evidence for its role as a facilitator of rejection and a regulator of tolerance. Created in BioRender. Wein, A. (2024) https://BioRender.com/r25f632.

In this article, we review the current literature regarding BALT and the work towards resolving its role in stimulating rejection versus inducing tolerance after lung transplantation (**Figure 1**). In doing so, we will discuss how insights gained from how BALT regulates immune responses to pulmonary allografts could be the basis for novel immunosuppressive therapies in lung transplant recipients.

## BALT as a facilitator of rejection

Early seminal studies examining the role of BALT in pulmonary transplantation were performed by Prop and colleagues in an orthotopic rat lung transplantation model (13–16). Prop and colleagues performed these studies to elucidate the mechanisms underlying pulmonary graft rejection, which they noted to be faster in tempo than that of other organs (13). Their use of a rat model is significant in that BALT is constitutive in rats, developing independently of microbial stimulation or immune activation, whereas healthy adult human lungs do not harbor BALT at baseline (7, 17, 18).

Prop and colleagues used Lewis (LEW), Brown Norway (BN), and F344 rats, which differ in their RT1 (rat major histocompatibility) alleles, along with the F1 generations LEW × BN and LEW × F344. Three combinations of allogeneic transplants, namely BN to LEW, LEW to BN, and F344 to LEW, and two semi-allogeneic transplants combinations, (LEW × BN) F1 to LEW and (LEW × F344) F1 to LEW, were employed. Left lungs were orthotopically transplanted without administration of antibiotics or immunosuppressants. The recipients were monitored for rejection by ventilation-perfusion scans and chest x-rays, with rejection being diagnosed as an observed decline in ventilatory function. Not surprisingly, rejection occurred more quickly in the allogeneic compared to the semi-allogeneic transplants. The authors found that eliminating graft lymphocytes in the BALT by irradiating the donors in the fully allogeneic combinations 24 hours before transplant prolonged graft survival compared to non-irradiated donor lungs but had negligible effect on the survival of semi-allogeneic transplants. Histologic analysis of the irradiated donor lungs confirmed that the BALT had been depleted of lymphocytes. However, dendritic cells, some of which reside in BALT, were notably radioresistant. In another set of experiments, the authors transplanted BN lungs into intermediate LEW recipients which had been treated with cyclosporine, then retransplanted these grafts into naive LEW hosts. This organ parking experiment allowed the exchange of graft cells with recipient cells of the first host, causing the grafts to lose immunogenicity by eliminating passenger cells including lymphocytes and dendritic cells. The retransplanted grafts subsequently demonstrated a longer survival than those in the radiation treatment group. From these findings, the authors concluded that lymphocyte aggregates and dendritic cells within the BALT of pulmonary grafts may be responsible for the accelerated rejection rate of lungs versus that of other transplanted organs.

Prop and colleagues sought to further elucidate the role of BALT in rejection and its interactions with donor and recipient lymphocytes by assessing the histology of grafts and various recipient tissues at various time points after transplantation (14). Two sets of RT1-incompatible (BN to LEW) lung transplants were performed along with syngeneic controls. In the first set of transplants, LEW rats were injected with chromium-51 labeled recipient-matched thoracic duct lymphocytes to allow assessment of graft infiltration by recipient cells. Recipients were sacrificed and the grafts were examined at various timepoints from 4 hours to 8 days after transplantation. The grafts demonstrated a progressive increase in recipient lymphocyte infiltration throughout the early post-operative course, from 0.58% of the LEW radiolabeled cells on post-operative day 1 to 3% of the radiolabeled cells on post-operative days 3 and 4. For comparison, syngeneic grafts or naïve lungs showed ~0.5% of radiolabeled recipient lymphocytes recruited in the absence of an allogeneic response. In the early phases of rejection, virtually all the radiolabeled recipient lymphocytes which infiltrated the graft were recruited to BALT sites, which demonstrated a proliferative immune response indicated by the presence of immunoblasts with high mitotic activity. In the second set of transplants, BN donors were injected with chromium-51 labeled donor-matched thoracic duct lymphocytes to label BALT lymphocytes in the graft and assess their dissemination into the recipient. Among these recipients, it was observed that most radiolabeled donor lymphocytes from the graft BALT had migrated to the spleens, livers, and lymph nodes of the recipient. Proliferative T and B cell responses were detected in the spleens. This work demonstrated a two-way lymphocyte exchange between the graft and recipient likely facilitated by the HEVs in BALT. The infiltrating recipient lymphocytes interact with BALT-resident immune cells, producing an *in situ* mixed leukocytes reaction which elicits a local rejection response, while donor leukocytes egressing from BALT disperse widely into recipient lymphoid tissues and stimulate systemic T cell activation and antibody production (14). Therefore, BALT was thought to contribute to both local and systemic immune processes that lead to graft rejection.

More recent work from The Toronto Lung Transplant Group investigated the impact of *de novo* lymphoid tissue on chronic allograft rejection (19). These authors obtained lung explants from thirteen patients diagnosed with BOS and compared them to fifteen control samples. Through immunofluorescence staining, they found that obliterative bronchiolitis (OB) and lymphocytic bronchiolitis (LB) lesions were associated with a higher number of HEVs, detected through staining for PNAd, whereas control lungs demonstrated a significantly smaller number of HEVs. The authors posited that the HEV-containing lymphoid structures they observed in lungs that had been chronically rejected were “effector lymphoid tissues.” To further clarify the role of *de novo* lymphoid tissue in graft rejection, the authors subsequently utilized a rat model involving orthotopic transplantation of lungs that bear intrapulmonary tracheal allografts. The authors observed the development of *de novo* lymphoid tissue around intrapulmonary tracheal allografts that their experiments suggested was able to propagate a local alloimmune response and thereby contribute to rejection. Notably, another study found that a decreased CD4/CD8 ratio in iBALT in human lung allografts is associated with episodes of acute cellular rejection (20).

More recently, a study using a syngeneic mouse lung transplant model with grafts that express diphtheria toxin (DT) receptor under the control of VEGFR3 allowing for inducible ablation of lymphatic vessels described a potential role for lymphatic disruption in the development of iBALT (21). DT administration 21 days after lung transplantation (a time point when lymphatic connections between the graft and the recipient have been re-established) resulted in loss of greater than 50% of lymphatic vessels as compared to non-treated control mice. The DT-treated mice developed iBALT within 5 days after treatment while control mice showed no iBALT development. The degree of lymphatic loss correlated with the number of iBALT structures. While the transplant model was not studied beyond 5 days, other experiments in this work suggested that the iBALT that develops after lymphatic disruption may be deleterious to lung function over longer time periods.

Thus, these studies gave rise to the notion that BALT may play an important role in acute and chronic rejection. While these studies in rat models were critical to improving our understanding of BALT’s role in pulmonary transplantation, there remains a key limitation in their clinical applicability: BALT is constitutive in rats and plays a key role in defense against pathogens, whereas BALT is largely induced in adult humans. Indeed, further studies utilizing transplant models in mice, in which – similar to humans – BALT is not constitutively expressed but can be induced following inflammatory stimuli, would suggest that the role of BALT in lung transplantation is more complex and can, in fact, contribute to immunological tolerance (6, 7).

## BALT as a regulator of tolerance

Informed by the findings of Prop’s rat lung transplant studies, Hasegawa and co-workers hypothesized that BALT would be associated with high-grade acute cellular rejection (ACR) and evidence of chronic rejection in human lung grafts (22). This group retrospectively examined the transbronchial biopsies of patients receiving single lung, double lung, and combined heart-lung transplants from 1984 to 1997 at a single institution. The group found that 77 patients had transbronchial biopsies demonstrating BALT. Contrary to the group’s hypothesis, in 75% of cases, the presence of BALT was associated with Grade 0 or 1 ACR, whereas only 9% of cases demonstrated the presence of iBALT in allografts with Grade 3 of ACR. Moreover, the percentage of BALT-positive biopsies demonstrating OB (29%), the pathological correlate of BOS, was not significantly different from that of the overall lung transplant population (34%) (22). Therefore, the authors inferred that BALT may induce tolerance in human lung transplantation, contrasting with the prevailing notion that BALT was a key mediator of rejection.

More recently, Snyder and colleagues followed twenty human leukocyte antigen (HLA) disparate lung transplant patients to study the persistence and role of donor-derived resident memory T cells in human lung allografts (23). Bronchoalveolar lavage (BAL) fluid showed high levels of donor CD4 and CD8 T cell persistence in all twenty patients up to 12 months following transplantation. However, examination of peripheral blood from fourteen recipients with samples at 3 or more timepoints found essentially no donor T cells in the peripheral blood by month 2 following transplantation. Both donor and recipient T cells within the BAL were predominantly resting effector memory T cells with no expression of the activation marker HLA-DR after one month post-transplant. Regulatory CD4^+^ T cells were decreased in the BAL and blood of transplant recipients compared to deceased donor control lungs and peripheral blood, presumably at least in part due to basiliximab infusion as part of conditioning. Immunofluorescence microscopy of three HLA-A2-disparate lung allografts showed that both donor and recipient T cells localized to BALT. While donor T cells were restricted to the BALT, recipient T cells were also found in other locations including peribronchiolar, subepithelial and intraepithelial regions. At all times, patients with ACR showed lower donor T cell chimerism, suggesting a protective role for these cells. These findings raise the possibility that BALT facilitates favorable immunological outcomes in humans by maintaining donor T cell chimerism.

Our group has investigated the function of BALT in pulmonary transplantation using an orthotopic mouse lung transplant model (24). We observed the induction of BALT in accepted lung grafts following treatment with peri-operative blockade of costimulatory pathways, a regimen that induces donor-specific tolerance. These structures contained T and B lymphocytes, PNAd^+^ HEVs, lymphatics and clusters of CD11c^+^ antigen presenting cells. Such structures were enriched with FoxP3^+^ regulatory T cells, which were often in close proximity to CD11c^+^ cells (25). These areas also showed a distinct appearance of blood vessels like those found in lymph nodes.

To examine the functional role of BALT-resident FoxP3^+^ T cells in maintaining tolerance, we used a re-transplant model (25). We observed that tolerant lung allografts maintain tolerance upon re-transplantation into non-immunosuppressed secondary recipients that are of the same genotype as the first recipient. These observations indicated that tolerogenic immune circuits are established locally in lung allografts. However, when FoxP3^+^ cells are depleted from the tolerant allograft at the time of re-transplantation into secondary hosts, we observed severe rejection (26). The rejection pattern has histological features characteristic of antibody-mediated rejection, including hyaline deposits, complement deposition, and airway epithelial damage. Furthermore, we observed development of donor-specific antibodies in the serum of these mice. Graft rejection was dependent on the ability of recipient B cells to produce antibodies, which may have been facilitated by their interaction with recipient T cells within the graft. Thus, BALT-resident FoxP3^+^ cells in tolerant lung allografts play a key role in downregulating humoral alloimmune responses locally. Subsequent work by our group demonstrated that BALT-resident FoxP3^+^ cells can downregulate alloimmune responses systemically in donor-specific fashion. Using lung re-transplantation models, we showed that FoxP3^+^ cells can leave BALT in tolerant lung allografts via lymphatics and promote the acceptance of cardiac allografts that are matched to the donor genotype of the lung graft. This finding extends observations of two human studies that reported an association between increased peripheral blood CD4^+^CD25^hi^CD127^low^ and FoxP3^+^ T cells and improved graft survival and decreased CLAD at two years after transplantation (27, 28).

A better understanding of mechanisms that contribute to the induction of BALT in tolerant lung grafts could lay the foundation for therapeutic approaches. In an allogeneic mouse lung transplant tolerance model, we showed that production of IL-22, primarily by recipient gamma-delta T cells and type 3 innate lymphoid cells, plays an important role in the induction of BALT (29). IL-22 is critical for the formation of PNAd^+^ HEVs and the recruitment of B cells to lymphoid follicles in tolerant lung allografts. However, we observed FoxP3^+^ cell aggregates even when recipients were IL-22-deficient, indicating that HEVs are not the only available route for FoxP3^+^ T cells to accumulate and cluster in tolerant lung allografts. Indeed, previous studies have shown that lymphocytes can enter BALT via an interstitial route when the HEV homing receptor L-selectin is inhibited (30).

To assess whether the presence of BALT in grafts impairs the ability to induce tolerance we induced BALT in donor mice through chronic exposure to cigarette smoking (31). This is a clinically relevant question as many transplant programs use lungs that could potentially harbor BALT due to a variety of inflammatory conditions. We found that the presence of BALT in donor lungs did not abrogate the ability to induce tolerance. In fact, we observed that tolerogenic FoxP3^+^ cell-enriched BALT formed at sites of pre-existing BALT, which may be consistent with Snyder’s observation of co-localization of donor and recipient immune cells within the same aggregates (23).

## Conclusions and future directions

While initial studies in rat lung transplant models suggested that BALT acts as a mediator of graft rejection, recent studies in mouse lung transplant models have raised the possibility that these structures, through accumulation of immunoregulatory cells, could play a significant role in dampening deleterious alloimmune responses locally and systemically. These findings in mouse lung transplant models align with some clinical observations that BALT may be protective against rejection in human pulmonary grafts. This may – at least in part – be explained by the fact that BALT is constitutive in rats and possibly primed to provoke a deleterious immune response due to the aggregation of antigen presenting cells. Conversely, BALT is induced in both mice and humans, and in the context of immunosuppression, alloreactive T cells may be prevented from expanding, which could facilitate the local accumulation of FoxP3^+^ regulatory T cells. These FoxP3^+^ cells may also regulate the function of local antigen presenting cells. One critical factor which may affect BALT in lung transplant patients is respiratory infection, which is a known risk factor for rejection. To this end, we and others have shown that *Pseudomonas aeruginosa* infection is associated with reduced CLAD-free survival (32). Given that BALT plays a key role in defense against respiratory pathogens, there is a possibility that its interactions with these pathogens may have a deleterious effect on its immunoregulatory functions in the graft.

The recent insights into iBALT’s importance in tolerance induction suggest a role for future therapies which promote FoxP3^+^ T cell aggregation and proliferation in grafts. There has already been considerable research devoted to developing immunotherapies which leverage FoxP3^+^ Tregs in the management of type I diabetes mellitus, systemic lupus erythematosus, and Crohn’s disease (33–35). In the realm of transplantation, there are nascent efforts to evaluate the viability of regulatory T cells in immunosuppression. Sanchez-Fuyeo demonstrated the safety of single-dose injections of autologous, polyclonally expanded Tregs as an adjunct to standard therapy in adult liver transplant patients (36). Similarly, Harden showed the safety of adjunct single-dose injections of FoxP3^+^ regulatory T cells in kidney transplant recipients (37). In the latter, significant and sustained expansion of regulatory T cells in the treated population was observed. At this time, such cellular therapies have not been trialed in the lung transplant population and may prove a fertile area for study. A recent report suggested a novel therapy for lung transplant patients. The Toronto Lung Transplant Group published a study of ex vivo expansion of regulatory T cells followed by infusion into the pulmonary circulation during normothermic ex vivo lung perfusion (EVLP) in rats (38). The expanded regulatory T cells maintained CD25 and FoxP3 expression and their administration did not affect measures of lung mechanics during EVLP. Histologic analysis of lungs transplanted following infusion of fluorescently labeled regulatory T cells showed the transferred cells outside of CD31^+^ blood vessels. Also, lungs from treated rats showed higher expression of regulatory T cell transcripts including *Foxp3* compared to non-infused control lungs. At 3 days after transplantation, treated lungs had increased numbers of regulatory T cells adjacent to antigen presenting cells compared to at the conclusion of EVLP. At this timepoint, treated lungs showed fewer conventional CD3^+^ T cells associated with antigen presenting cells compared to nontreated lungs. In addition, conventional CD3^+^ T cells showed less expression of activation markers including ICAM1 and CD44 compared to control lungs.

In conclusion, there is an urgent need to develop immunosuppressive therapy that accounts for the unique immunological characteristics of lungs, especially since recent studies have suggested that current therapies may interrupt tolerogenic pathways. For example, the use of basiliximab as induction therapy in lung transplantation may adversely impact the ability of regulatory T cells to expand in the graft. Consideration of the unique immune environment in the lung and development of specific immunosuppression regimens for lung transplantation is required to extend graft survival.
